# Comparison of Sensation Event Descriptors in Participants with Overactive and Normal Bladders during Non-Invasive Hydration Studies

**Published:** 2021-11-26

**Authors:** Blessan Sebastian, Natalie R. Swavely, Dhruv Sethi, Anna S. Nagle, Devina Thapa, Naomi N. Vinod, Zachary E. Cullingsworth, Andrea K. Balthazar, Adam P. Klausner, John E. Speich

**Affiliations:** 1 Department of Surgery/Division of Urology, Virginia Commonwealth University School of Medicine, Richmond, VA, USA; 2 Department of Mechanical and Nuclear Engineering, Virginia Commonwealth University College of Engineering, Richmond, VA, USA

**Keywords:** Diuresis/physiology, Sensation/physiology, Urinary Bladder/physiology, Overactive bladder

## Abstract

**Purpose:**

Despite the importance of alterations in bladder sensation, objective metrics to characterize sensation outside of urodynamics remain limited. A real-time sensation meter enables recording of sensation event descriptors throughout filling. The purpose of this study was to evaluate the differences in sensation event descriptor patterns between normal participants and those with OAB.

**Methods:**

Normal and OAB participants were enrolled from responses to the ICIq-OAB survey question on urgency (Q5a: 0 vs. ≥ 3). Real-time bladder sensation on a 0%-100% scale was recorded on a validated tablet sensation meter throughout two fill-void cycles. The first and second fills were considered “slow” and “fast” respectively. After each sensation meter change (sensation event), a pop-up screen asked participants to characterize sensation with one or more of these descriptors: “tense,” “pressure,” “tingling,” “painful,” and/or “other.” Oral hydration was achieved by rapid consumption of 2L G2^®^ Gatorade.

**Results:**

Data from 29 participants (12 normal/17 OAB) were analyzed. The rate of filling from bladder volume and fill duration, was greater for the fast fill in both groups. In the slow fill, “tingling” (64 ± 3% OAB vs. 77 ± 3% normal, p=0.008) and “tense” (78 ± 3% OAB vs. 94 ± 1% normal, p<0.001) occurred at lower sensations in OAB participants.

**Conclusion:**

During only the slow fill, OAB individuals experience the sensation descriptors of “tingling” and “tense” at earlier sensations than normal individuals. Therefore, this non-invasive method to evaluate real-time sensation descriptors during filling may identify important sensation patterns and improve understanding and phenotyping of OAB.

## Introduction

Overactive bladder (OAB) is a syndrome defined by the sensation of urinary urgency [1]. According to the International Continence Society, OAB can be associated with incontinence, urinary frequency, and nocturia [1]. OAB is a highly prevalent condition with an estimated 30 million adults over the age of 40 with some degree of OAB symptoms [2]. Due to the significant impact on life quality, patients spend a considerable amount of health care dollars on treatment of OAB symptoms [3].

As OAB syndrome is defined by the feeling of urgency. The hallmark of this syndrome is aberrations in bladder sensation. However, standardized methods to evaluate real-time bladder sensation beyond conventional urodynamics are not widely available. To address this, a previously engineered tablet-based sensation meter was developed and tested. This sensation meter enables recording of real-time changes in sensation and unique sensation event descriptors during non-invasive oral hydration [4-6]. The sensation event descriptors of “pressure”, “tingling”, “tense” and “painful” were developed in focus group introspection studies [7,8] and evaluated during oral hydration studies in normal participants [4,6].

The previously published study in normal participants identified unique sensation event descriptor patterns including (1) the consistent identification of 8-9 sensation events per fill, (2) the finding that perception of sensation events occurs mainly after 50% sensation, and (3) the finding that “pressure” and “tingling” were the most commonly chosen sensation event descriptors [6]. Therefore, the purpose of the current study was to characterize and quantify differences in real-time bladder sensation event descriptors between OAB versus normal individuals during a similar non-invasive oral hydration protocol. Event-Descriptor patterns could ultimately be used in future studies to help improve the phenotyping and treatment of OAB.

## Materials and Methods

This prospective study was approved by our Institutional Review Board. Using the validated ICI-q OAB questionnaire, patients with normal bladders (ICIq OAB question 5a=0 and all other questions ≤ 1) and OAB (ICIq question 5a ≥ 3 with any other question responses) were enrolled. Participant characteristics including age, gender, race, Body Mass Index (BMI), comorbidities, and medication use were obtained from all participants. In addition, participants were excluded if they had any known or suspected neurologic conditions that could affect bladder sensation. Participants viewed a standardized training video before study initiation on the sensation meter and were allowed to ask questions preceding the study about sensation meter use.

### Sensation meter

**Figure 1:**
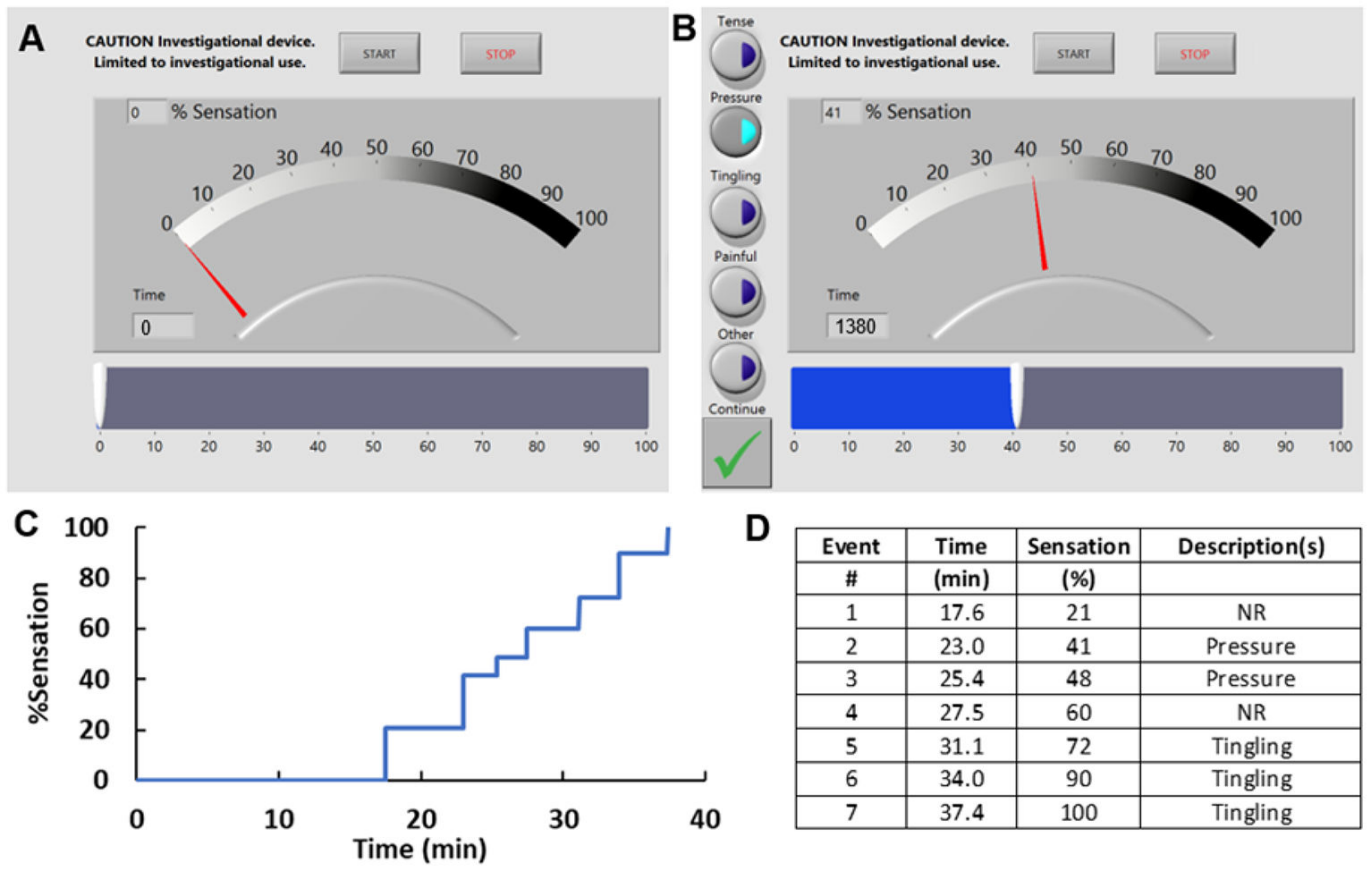
1A: Screenshot of the tablet sensation meter showing the participant interface (blue slide bar). 1B: Screenshot showing the pop-up menu of sensation event descriptors (left) which occurs after the level of sensation is changed. 1C: Example sensation-time graph for a participant where each change in sensation (blue line) is noted by a new step. 1D: Example of sensation event descriptors recorded for every sensation event in the same participant.

The sensation meter is a touch-screen tablet (Surface Pro3, Microsoft, Redmond, WA) on which LabView (National Instruments, Austin, TX) software created a user interface that allows the participant to quantify bladder sensation on a scale from 0%-100%. Participants were instructed to adjust the percentage of bladder sensation as they perceived changes in bladder fullness sensation throughout the study (Figure 1A). Participants were not prompted by study personnel. Each sensation meter adjustment was defined as a “sensation event.” With each sensation event, a pop-up menu prompted participants to characterize the event with the following descriptors: “tense,” “pressure,” “tingling,” “painful,” and/or “other” (Figure 1B). After study completion, participants completed a survey on the ease of use and meter understanding.

### Hydration protocol

All participants completed a two-fill hydration protocol based on previously published methods [6] with some modifications. Upon arrival, participants were asked to void, and a Post-Void Residual (PVR) was obtained using BladderScan (Verathon, Seattle, Washington). After voiding, the sensation meter was started, and participants were immediately instructed to drink 2L G2^®^ Gatorade as quickly as comfortably possible. G2^®^ Gatorade was chosen as a low-sugar drink to prevent the rare risk of water intoxication. During filling, participants underwent bladder ultrasound to assess bladder volume at ten-minute intervals. Once participants reached 100% sensation, they were escorted to a private bathroom to void into a urinal/hat. Voided volume was measured and PVR was again obtained. The total filling volume was considered the voided volume plus PVR. When participants returned, the sensation meter was reset to 0 for the second fill, and they were instructed to consume an additional volume of G2^®^ Gatorade equal to the voided volume to ensure maximal diuresis was maintained during fill 2 following published protocols [8]. Once participants again reached 100% sensation, the procedure was repeated, providing sensation data for two fill-void cycles where fill 1 was defined as a “slow fill” and fill 2 was defined as a “fast fill.”

### Sensation events

Throughout the two fill-void cycles collected data included: Voided volumes, PVR, fill time, percent sensation (0%-100%), and sensation event descriptors (“tense,” “pressure,” “tingling,” “painful,” and “other”). Sensation events were recorded when the meter was moved to a new sensation level for at least 10 seconds based on previous methods [6]. Additionally, volume and percent capacity were estimated by determining the average fill rate by dividing total filling volume by fill duration, separately for the slow and fast fill [4,5]. Example participant data is shown in Figures 1C and 1D.

### Statistical analysis

Bivariate comparisons were assessed with students’ t-test and multivariate comparisons were assessed with ANOVA. All data are reported as mean ± standard error. Post-hoc power analysis demonstrated a sample size of at least 12 was needed to achieve a power of 0.80.

## Results

Twenty-nine participants completed the protocol, including 12 participants with normal bladder sensation (ICIq question 5a=0: Never having to rush to the toilet to urinate) and 17 high urge OAB participants (question 5a ≥ 3: Having to rush to the toilet to urinate most of the time/all the time). Participant information regarding age, biological sex, and BMI is listed in Table 1. These variables were not statistically significantly different between the two groups (p>0.05).

Participant’s total bladder volume was calculated by combining the amount voided to the post-void bladder scan amount (PVR). For the slow fill, this was 592.8 ± 104.2 mL for normal participants and 461.1 ± 70.9 mL for OAB participants. For the fast fill, this was 652.5 ± 96.1 mL and 501.5 ± 59.9 mL for normal and OAB respectively. There were no statistically significant differences found in total bladder volume between normal and OAB participants for either fill, and there was no difference in the fills for either participant group for total bladder volume (p>0.05). There was also no difference in PVR between participant groups for fills or amongst participant groups (p>0.05).

The slow fill duration for the normal group was 87.6 ± 7.2 minutes and 78.6 ± 9.3 minutes for the OAB group. For the fast fill, this was 55.2 ± 5.6 minutes and 38.1 ± 5.9 minutes for the normal and OAB groups respectively. The bladder filling rate was not statistically different between normal vs. OAB participants; However, both groups had a significant increase in rate during the fast fill. The filling rate for the slow fill in the normal group was 6.3 ± 0.9 vs. 6.8 ± 0.9 mL/min in the OAB group (p>0.05), and the fast filling rate was 11.9 ± 1.4 vs.15.0 ± 1.4 in the normal and OAB groups, respectively (p>0.05). These rates demonstrate the fast fill is approximately 2x faster than the slow fill for both normal (p<0.001) and OAB (p<0.001) groups.

**Figure 2:**
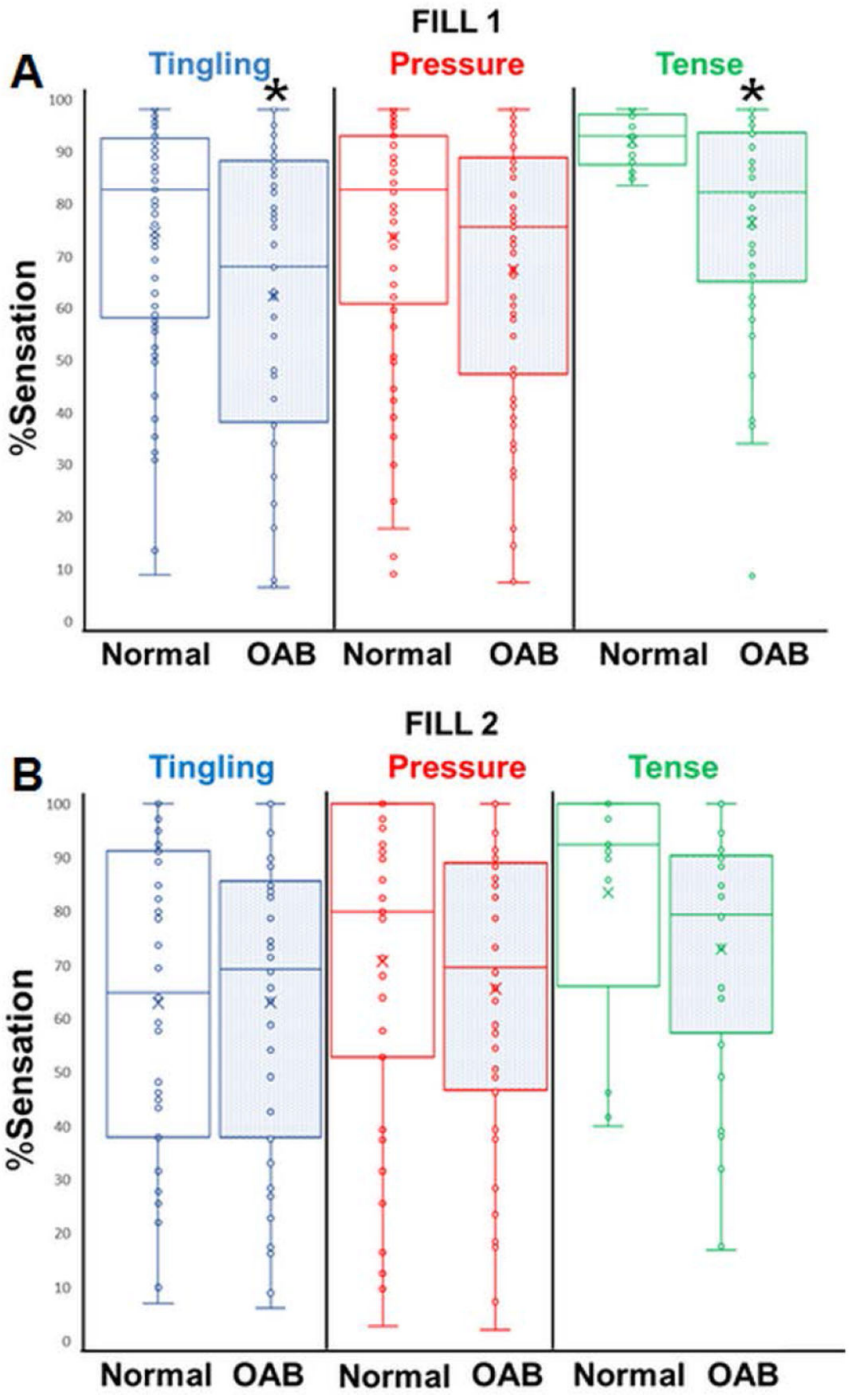
Box-and-whisker plots of percent sensation for each reported sensation event descriptor. Shaded plots represent the OAB group results. **Note:** (*) Indicates statistical significance when compared to the same descriptor in the healthy group.

In the slow fill, sensation events of “tingling” (64.1 ± 3.4% OAB vs. 76.5 ± 3.0% normal, p=0.008) and “tense” (78.4 ± 3.1% OAB vs. 93.9 ± 1.4% normal, p0.05), (Figure 2A, Table 2). Contrastingly, in the fast fill, there were no differences in bladder sensation percentage for any event descriptors. (Figure 2B, Table 2). “Painful” was not compared between groups as it was only rarely selected at sensations >90% in normal participants and >70% in OAB participants in both fills (Table 2).

When comparing the slow to fast fill, OAB participants exhibited no difference in percentage sensation for any descriptors (Table 3). In contrast, normal sensation participants reported tingling in the fast fill at 64.1 ± 4.1% vs. 76.5 ± 3.0% in the slow fill (p=0.02). Normal participants did not exhibit any other statistically significant differences between other descriptors for fast and slow fills (Table 3).

The number of sensation events was compared between OAB and normal participants. For the slow fill, OAB participants recorded 9.9 ± 1.0 sensation events and normal participants reported 13.1 ± 1.8 events (p=0.04). However, this difference was not seen in the fast fill (OAB: 8.3 ± 0.7 events vs. normal: 10.8 ± 1.2 events, p=0.07). When comparing slow fill to fast fill, normal participants did not show a difference (p=0.06); however, OAB participants had a significant decrease (p=0.04).

## Discussion

This study demonstrates that a sensation meter can be used to quantify bladder filling sensations during a non-invasive hydration protocol and that OAB patients experience different sensation patterns than people with normal bladders. The current investigation findings are consistent with previous publications that pressure and tingling are commonly felt sensations, and that painful is a relatively infrequent sensation [5-8]. A real-time sensation meter offers a more objective method of quantifying bladder sensation as it removes recall bias seen with conventional bladder diaries [5,6,9].

In this study, OAB participants reported sensation event descriptors of “tingling” and “tense” at lower sensation percentages than normal participants. Thus, these descriptors may be important cognitive triggers for urination at lower volumes, a hallmark of OAB. Additionally, this study showed fast filling can lead normal individuals to experience OAB sensations because sensation event patterns in normal participants during fast filling were similar to OAB participants during slow filling, which provides a potential explanation for why otherwise normal individuals who consume large quantities of fluids may experience urgency with frequency.

Interestingly, patterns of sensation event descriptors in OAB participants were not affected by fill rates, as there was no difference in any descriptors between slow and fast fills. However, there was a decrease in total number of sensation events with faster filling. This suggests decreased warning (i.e. fewer events) and highlights the complexity involved in the interpretation of sensation which likely involves fill rate variables and the ability of detrusor smooth muscle to function as an intrinsic tension sensor. Studies provide evidence for in-series A-delta tension sensors in the bladder [10]. Clinically, this data could be important when considering fill rates for both urodynamics and hydration protocols in bladder function evaluation. Sensation interpretation is even more complex when considering that urodynamic studies have shown super physiologic fill rates result in higher fill volumes, which would suggest an impaired warning mechanism. This is consistent with the reduced number of sensation events in OAB participants seen in the current study [11].

The number of sensation events reported was less in the OAB group than the normal group for the slow, but not fast fill. One possible explanation is the OAB group likely had fewer sensation events because they approached 100% sensory capacity quicker. During the fast fill, which we theorized may induce OAB behavior in normal patients, there is no longer a difference in sensation event numbers between OAB and normal groups. This difference in the OAB group is in contrast to our previously published results in normal participants that did not show a difference in sensation events numbers between slow and fast fills [6]. However, because the data approached significance (p=0.06), this may reflect lower numbers of normal participants in the current study.

Several investigators have attempted to improve metrics for bladder sensation. Lowenstein and colleagues [12] developed an “urgometer” which was a manual rheostat device providing real-time sensation data directly incorporated into commercially available UDS equipment. Data from this study demonstrated differences in sensation patterns between women with different types of urinary incontinence (stress, urge, or mixed). However, no further studies were published, and the device is not commercially available. Another method for objective bladder sensation characterization includes electrical sensory perception threshold testing, [13,14] but this is invasive, requiring bladder catheterization. The current gold standard for objective bladder sensation characterization is multi-channel urodynamics in which patients are prompted to report standardized verbal sensory thresholds of first sensation of filling, first desire to void, and strong desire to void [1]. However, this type of testing can be affected by investigator prompting and non-physiologic, artificial nature of testing [15,16].

Limitations in this study include a single-institution study with a relatively small sample size. Additionally, the average age of this participant group was young with normal sensation patients having an average age of 24.6 ± 1.2 and the OAB group 26.8 ± 2.3. There was also a significantly increased number of women in the OAB group compared to men (13F/4M). This is reflective of the typical OAB population which includes a higher percentage of women [2]. Additionally, participants underwent ultrasound examinations throughout the protocol, and external pressure from these examinations may have affected bladder sensation [4].

Additional randomized studies with larger sample sizes to further quantify the sensations experienced by individuals with OAB. These studies will be necessary to provide predictive analytics that will hopefully be used to improve the way we diagnose and treat OAB. Once established as a clinical tool, the sensation meter could be adapted to a mobile phone application enabling bladder sensation monitoring in a truly physiologic setting.

## Conclusion

These results suggest that OAB participants report the sensation event descriptors of “tingling” and “tense” at lower percentage bladder sensations than normal participants during slow filling. These results also suggest that normal participants may experience OAB-type sensation event patterns during fast filling. The use of a real-time sensation meter with sensation event descriptors represents a non-invasive method to improve understanding of bladder sensation, and sensation event patterns may allow for improved phenotyping of OAB.

## Figures and Tables

**Table 1:** Participant information.

Group	Normal	OAB	p-value
Age	24.6 ± 1.2	26.6 ± 2.2	>0.05
Biological sex	6M/6F	4M/13F	>0.05
BMI	23.3 ± 0.7 kg/m^2^	23.7 ± 1.1 kg/m^2^	>0.05

**Table 2:** OAB vs. Normal^*^

Slow fill	OAB	Normal	p-value
Tense	78.4 ± 3.1%	93.9 ± 1.4%	<0.001
Pressure	69.3 ± 2.9%	75.5 ± 3.4%	0.17
Tingling	64.1 ± 3.4%	76.5 ± 3%	0.008
Painful	90.1 ± 2.8%	97.6 ± 2%	0.053
Fast fill	OAB	Normal	p-value
Tense	73.8 ± 4.6%	83.9 ± 6.4%	0.21
Pressure	66.7 ± 3.6%	71.6 ± 5%	0.43
Tingling	64.3 ± 3.9%	64.1 ± 4.1%	0.98
Painful	82.5 ± 4.1%	-----	----

Note:

* Average percentage sensation at which event descriptors occur.

**Table 3:** Slow vs. Fast fill^*^.

OAB group	Slow fill	Fast fill	p-value
Tense	78.4 ± 3.1%	73.8 ± 4.6%	0.41
Pressure	69.3 ± 2.9%	66.7 ± 3.6%	0.57
Tingling	64.1 ± 3.4%	64.3 ± 3.9%	0.98
Painful	90.1 ± 2.8%	82.5 ± 4.1%	0.16
Normal group	Slow fill	Fast fill	p-value
Tense	93.9 ± 1.3%	83.9 ± 6.4%	p=0.14
Pressure	75.5 ± 3.4%	71.6 ± 5%	p=0.52
Tingling	76.5 ± 3.0%	64.1 ± 4.1%	p=0.02
Painful	97.6 ± 2.0%	-----	-----

Note:

* Average percentage sensation at which event descriptors occur.
